# Evaluating functional disability in clinical trials of lisdexamfetamine dimesylate in binge eating disorder using the Sheehan Disability Scale

**DOI:** 10.1002/mpr.1849

**Published:** 2020-08-25

**Authors:** Karen S. Yee, Robin Pokrzywinski, Asha Hareendran, Shannon Shaffer, David V. Sheehan

**Affiliations:** ^1^ Shire a Member of the Takeda Group of Companies Cambridge Massachusetts USA; ^2^ Patient‐Centered Research Evidera Bethesda Maryland USA; ^3^ Patient‐Centered Research Evidera London UK; ^4^ University of South Florida College of Medicine Tampa Florida USA

**Keywords:** binge eating disorder, psychometric properties, reliability, responsiveness, validity

## Abstract

**Objectives:**

This study examined Sheehan Disability Scale (SDS) performance in binge eating disorder (BED) and explored relationships between SDS and BED outcomes using data from three placebo‐controlled lisdexamfetamine (LDX) studies (two short‐term, dose‐optimized studies and one double‐blind, randomized‐withdrawal study) in adults with Diagnostic and Statistical Manual of Mental Disorders, fourth edition, text revision (DSM‐IV‐TR)–defined BED.

**Methods:**

Analyses evaluated the psychometric properties of the SDS.

**Results:**

Confirmatory factor analysis supported a unidimensional total score in the short‐term studies, with internal consistency (Cronbach's *α*) being 0.878. Total score exhibited good construct validity, with moderate and statistically significant correlations observed with Yale–Brown Obsessive Compulsive Scale modified for binge eating, Binge Eating Scale (BES), and EuroQol Group 5‐Dimension 5‐Level health status index scores. Known‐groups validity analysis for the short‐term studies demonstrated a significantly lower total score at end of study in participants considered “not ill” versus “ill” based on Clinical Global Impressions–Severity scores. SDS total score changes in the short‐term studies were greater in responders than nonresponders based on binge eating abstinence or BES score. In the randomized‐withdrawal study, SDS scores increased relative to baseline to a greater extent in participants randomized to placebo than LDX.

**Conclusions:**

These analyses support the reliability, validity, and responsiveness to change of the SDS in individuals with BED.

**Abbreviations**
ADHD: attention‐deficit/hyperactivity disorder
ANCOVA: analysis of covariance
BED: binge eating disorder
BES: Binge Eating Scale
CI: confidence interval
CFI: comparative fit index
CGI‐S: Clinical Global Impressions–Severity
CGI‐I: Clinical Global Impressions–Improvement
DF: degrees of freedom
EOS: end of study
EQ‐5D‐5L: EuroQol Group 5‐Dimension 5‐Level
ES: effect size
LDX: lisdexamfetamine dimesylate
N/A: not applicable
OL: open label
RMSEA: root‐mean‐square error of approximation
ROC: receiver operating characteristic
RWB: randomized‐withdrawal baseline
SD: standard deviation
SDS: Sheehan Disability Scale
SEM: standard error of the mean
SRMR: standardized root mean residual
VAS: visual analogue scale
Y‐BOCS‐BE: Yale–Brown Obsessive Compulsive Scale modified for binge eating
YI: Youden's index

## 1 INTRODUCTION

Individuals with binge eating disorder (BED) experience impaired function across multiple domains (Johnson, Spitzer, & Williams, 2001; Kessler et al., 2013). In an analysis of the World Health Organization World Mental Health Survey (Kessler et al., 2013), impairment on the Sheehan Disability Scale (SDS) was reported in 46.7% of individuals with BED. In another study, women with BED exhibited poor physical, social, and role functioning; poor mental health; and poor general health perception on the Medical Outcomes Study Short‐Form General Health Survey (Johnson et al., 2001).

The SDS, which has been used to assess functional impairment across multiple psychiatric disorders (Arbuckle et al., 2009; Coles, Coon, DeMuro, McLeod, & Gnanasakthy, 2014; Jacobsen, Mahableshwarkar, Serenko, Chan, & Trivedi, 2015; Leon, Shear, Portera, & Klerman, 1992; D. V. Sheehan et al., 2011; Weiss et al., 2012), assesses impairment in the domains of work/school, social life/leisure activities, and family life/home responsibilities (Leon, Olfson, Portera, Farber, & Sheehan, 1997; Rush, First, & Blacker, 2008; K. H. Sheehan & Sheehan, 2008). The psychometric properties of the SDS have been explored in multiple psychiatric conditions (Arbuckle et al., 2009; Coles et al., 2014; Leon et al., 1992), but not in individuals diagnosed with BED. Among adults diagnosed with attention‐deficit/hyperactivity disorder (ADHD), the SDS demonstrated statistically significant inter‐item correlations at baseline (correlation coefficients: 0.23–0.62, all *p* < 0.001) and week 9 (correlation coefficients: 0.38–0.79, all *p* < 0.001), good internal consistency (Cronbach's α of 0.79 at baseline and 0.91 at week 9), and known‐groups validity (Coles et al., 2014). The SDS exhibited a single‐factor structure and demonstrated strong item‐total score correlations (correlation coefficients: 0.77–0.80), good internal consistency (Cronbach's *α*, 0.89), and known‐groups validity in adults diagnosed with bipolar disorder (Arbuckle et al., 2009).

This report describes the performance of the SDS in individuals diagnosed with BED and explores the relationships between SDS scores and BED outcomes using data from Phase 3 lisdexamfetamine dimesylate (LDX) clinical studies (Hudson, McElroy, Ferreira‐Cornwell, Radewonuk, & Gasior, 2017; McElroy et al., 2016). In two short‐term, Phase 3, randomized, placebo‐controlled efficacy studies (McElroy et al., 2016), LDX reduced binge eating days/week (primary endpoint) in adults diagnosed with moderate to severe BED and was associated with greater reductions in SDS scores than placebo (D. V. Sheehan et al., 2018). In a maintenance‐of‐efficacy study, LDX treatment was associated with longer time to relapse (primary endpoint) to binge eating over 6 months compared with placebo (Hudson et al., 2017). The SDS was included as a secondary endpoint in this study, but the findings for the SDS have not been described.

## 2 METHODS

Detailed descriptions of the study designs and participants for these trials have been reported (Hudson et al., 2017; McElroy et al., 2016). A brief summary is provided here.

### 2.1 Study design and treatment

Two identically designed, randomized, placebo‐controlled, parallel‐group, multicenter short‐term trials (http://clinicaltrials.gov/ identifiers: NCT01718483 [conducted in the United States, Germany, Spain, and Sweden] and NCT01718509 [conducted in the United States and Germany]) and one double‐blind, placebo‐controlled, maintenance‐of‐efficacy trial (http://clinicaltrials.gov/ identifier: NCT02009163 [conducted in the United States, Germany, Sweden, Spain, and Canada]) were used for these analyses. Study protocols were approved by ethics committees. Each study was conducted in accordance with the International Council for Harmonization Good Clinical Practice and the principles of the Declaration of Helsinki. Participants provided written‐informed consent before study‐related procedures were conducted.

The short‐term studies included 3 phases: a 2‐week screening phase, a 12‐week double‐blind phase (4 weeks of dose optimization and 8 weeks of dose maintenance), and follow‐up. Participants were randomized 1:1 to receive 12 weeks of dose‐optimized LDX (50 or 70 mg) or matching placebo. Treatment began with 30 mg LDX during week 1. At the start of week 2, the LDX dose was increased to 50 mg. During week 3, the LDX dose was increased to 70 mg based on tolerability and clinical need. A single‐dose reduction from 70 to 50 mg was allowed during week 3 if tolerability was poor; no additional dose changes were allowed if such a reduction occurred. During dose maintenance, the optimized LDX dosage was maintained. No dose changes were permitted beyond week 3; any participant requiring a dose reduction during the maintenance phase was discontinued. A follow‐up visit occurred 1 week after the final treatment visit to assess ongoing or new safety/tolerability issues.

The maintenance‐of‐efficacy study included a 12‐week, open‐label dose‐optimization phase (4 weeks of dose optimization and 8 weeks of dose maintenance); a 26‐week, double‐blind, randomized‐withdrawal phase; and a 1‐week follow‐up phase (Hudson et al., 2017). During the open‐label dose‐optimization phase, participants started treatment with 30 mg LDX during week 1. During weeks 2 and 3 of dose optimization, the LDX dose increased to 50mg and then 70 mg, respectively. A dose reduction to 50 mg LDX was allowed if 70 mg LDX was not tolerated; however, once a dose reduction occurred, further changes were not allowed. No dose changes were permitted after week 3. Participants who could not tolerate 50 mg LDX were discontinued. At the end of open‐label treatment, participants categorized as LDX responders (i.e., those reporting ≤1 binge eating day/week for four consecutive weeks and having a Clinical Global Impressions–Severity [CGI‐S] rating ≤2) were randomized 1:1 to placebo or continued LDX treatment (50 or 70 mg) at their established dose‐optimized level.

### 2.2 Participants

Across studies, eligible participants were adults (18–55 years), met *Diagnostic and Statistical Manual of Mental Disorders, Fourth Edition, Text Revision* criteria for BED, and had protocol‐defined moderate to severe BED (≥3 binge eating days/week for 14 days before baseline [open‐label baseline in the maintenance‐of‐efficacy study] and a CGI‐S rating ≥4 at screening and baseline [open‐label baseline in the maintenance‐of‐efficacy study]). Participants were also required to have body mass index ≥18 and ≤45 kg/m^2^ at screening and baseline (open‐label baseline in the maintenance‐of‐efficacy study) and to provide written‐informed consent.

Study exclusion criteria included a current diagnosis of anorexia nervosa or bulimia nervosa, current comorbid psychiatric disorder controlled with prohibited medications or uncontrolled with significant symptoms, or condition that may confound study assessments. Participants were not permitted to receive psychotherapy or weight loss support for BED ≤3 months before screening; have a Montgomery‐Åsberg Depression Rating Scale total score ≥18 at screening; or be considered a suicide risk, have previously attempted suicide, or be currently demonstrating active suicidal ideation. Having a history of symptomatic cardiovascular disease, structural cardiac or heart rhythm abnormalities, or moderate or severe hypertension, or average sitting systolic blood pressure >139 mmHg, or average diastolic blood pressure >89mmHg at screening or baseline were also exclusionary. Participants with a lifetime history of stimulant abuse, a history of substance abuse or dependence within the past 6 months or known or suspected intolerance or hypersensitivity to LDX or related compounds were also excluded.

### 2.3 Measures

Functional disability across the SDS domains (work/school, social life/leisure activities, and family life/home responsibilities) was assessed at baseline, week 6, and week 12 in the short‐term studies, and at open‐label baseline (day 0) and weeks 4, 12/randomized‐withdrawal baseline, 16, 20, 24, 28, 32, and 38 in the maintenance‐of‐efficacy study. Item responses were scored on a discretized‐analogue (Discan) metric (0 [not at all] to 10 [extremely]). Item scores were summed to generate a total score (range: 0 [unimpaired] to 30 [extremely impaired]; K. H. Sheehan & Sheehan, 2008).

Measures of binge eating included the Yale–Brown Obsessive Compulsive Scale modified for Binge Eating (Y‐BOCS‐BE; Deal, Wirth, Gasior, Herman, & McElroy, 2015), the Binge Eating Scale (BES; Gormally, Black, Daston, & Rardin, 1982), and binge eating frequency based on self‐report diary entries. The Y‐BOCS‐BE, a 10‐item clinician‐rated scale that assesses the obsessiveness of binge eating thoughts and compulsiveness of binge eating behaviors (Deal et al., 2015), was conducted at baseline and at weeks 4, 8, and 12 in the short‐term studies, and at open‐label baseline and weeks 4, 12/randomized‐withdrawal baseline, 16, 20, 24, 28, 32, and 38 in the maintenance‐of‐efficacy study. Individual items were scored on a scale from 0 (no symptoms) to 4 (extreme symptoms) and summed to generate a total score (range: 0–40). The BES, which was used only in the short‐term studies, is a 16‐item self‐report questionnaire that assesses the behavioral, affective, and attitudinal components of binge eating (Gormally et al., 1982; Timmerman, 1999). The BES was assessed at baseline and at weeks 4, 8, and 12. Items were scored on scales ranging from 0 (no binge eating problem) to 3 (severe binge eating problem). Total score ranges from 0 to 46 (Timmerman, 1999), with scores ≤17 indicating little or no binge eating (Marcus, Wing, & Lamparski, 1985). Binge eating days/week was recorded daily in self‐report diaries; entries were reviewed with the participant and confirmed by study investigators at each study visit.

Overall BED severity and its improvement over time were assessed with the 7‐item, clinician‐rated CGI‐S and Clinical Global Impressions–Improvement (CGI‐I) scales, respectively (Guy, 1976). The CGI‐S rates the severity of a participant's condition (range: 1 [normal, not at all ill] to 7 [among the most extremely ill]). The CGI‐I rates improvement in the participant's condition relative to baseline (range: 1 [very much improved] to 7 [very much worse]). The CGI‐S was administered at all visits; the CGI‐I was administered at all visits except screening and baseline in the short‐term studies and except screening and follow‐up in the maintenance‐of‐efficacy study.

Quality of life, which was measured using the EuroQol Group 5‐Dimension 5‐Level (EQ‐5D‐5L) scale was assessed at baseline and at weeks 4, 6, 8, 10, and 12 in the short‐term efficacy studies and at screening, open‐label baseline, and weeks 4, 12/randomized‐withdrawal baseline, and 38 in the maintenance‐of‐efficacy study. The EQ‐5D‐5L is a self‐report scale that measures 5 dimensions of quality of life (mobility, self‐care, usual activities, pain/discomfort, and anxiety/depression; Herdman et al., 2011; Janssen et al., 2013) using five response levels (no problems to extreme problems); individual dimension responses can be combined to generate a health status index. A visual analogue scale (VAS) is used to record self‐rated health, with endpoints recorded as “the best health you can imagine” (score = 100) and “the worst health you can imagine” (score = 0).

### 2.4 Endpoints

The prespecified efficacy, safety, and tolerability findings from these studies have been published (Hudson et al., 2017; McElroy et al., 2016). These post hoc analyses were conducted in the full analysis set (short‐term studies: randomized participants taking ≥1 study drug dose and having ≥1 postbaseline primary efficacy assessment; maintenance‐of‐efficacy study: randomized participants who took ≥1 study drug dose during the randomized‐withdrawal phase and who had ≥1 postrandomization CGI‐S assessment). Data from the short‐term studies were pooled.

For the short‐term studies, data from baseline (binge eating days/week, Y‐BOCS‐BE, EQ‐5D‐5L, CGI‐S, BES, and SDS), week 6 (SDS and CGI‐S), and end of study (EOS) (SDS, BES, CGI‐S, and CGI‐I) were used. EOS was defined as week 12 for the SDS and BES (only observed cases were used) and as week 12/early termination, with last observation was carried forward if week 12 data were missing, for the CGI‐S and CGI‐I. For the maintenance‐of‐efficacy study, data from screening (binge eating days/week, Y‐BOCS‐BE, EQ‐5D‐5L, CGI‐S, and SDS), open‐label baseline (binge eating days/week, Y‐BOCS‐BE, EQ‐5D‐5L, CGI‐S, and SDS), week 12/randomized‐withdrawal baseline (SDS), week 16 (SDS), and EOS (SDS) were used. EOS was defined as week 38/early termination. Descriptive statistics for baseline sociodemographic variables are presented.

### 2.5 Data presentation and analyses

Psychometric analyses determined the factor structure, reliability, validity, and responsiveness to treatment of the SDS. Across all analyses, significance level of *p* < 0.05 was used.

Item‐level analyses examined SDS scores and reference measures at baseline in the short‐term studies and at open‐label baseline in the maintenance‐of‐efficacy study using descriptive statistics. SDS score response distributions were examined for floor and ceiling effects (having >30% of responses in the minimum or maximum response categories) at baseline and EOS in the short‐term studies, and at open‐label baseline and randomized‐withdrawal baseline in the maintenance‐of‐efficacy study. Confirmatory factor analyses were conducted at baseline in the short‐term studies and at open‐label baseline in the maintenance‐of‐efficacy study using two models (constraining factor loadings for the social life and family life domains to be equal [primary analysis] and constraining the measurement error variances to be equal [secondary sensitivity analysis]) to assess the underlying structure of SDS total score. Fit statistics included the comparative fit index (acceptable values, ≥0.9), standardized root mean residual (acceptable values, <0.1), and root mean square error of approximation (acceptable values, <0.08); factor loadings ≥0.40 were considered acceptable.

Reliability was assessed with internal consistency and test–retest reliability in the short‐term studies. Internal consistency was assessed at baseline for SDS total and domain scores, with Cronbach's α values ranging from 0.70 to 0.90 being considered acceptable (Streiner & Norman, 1995). Test–retest reliability examined reproducibility over time under stable clinical conditions. These analyses, which were conducted in participants receiving placebo who had the same CGI‐S rating at baseline and week 6, calculated intraclass correlation coefficients and change scores between baseline and week 6. It was hypothesized that SDS scores would not be statistically different, as measured using two‐sided paired *t*‐tests between the two time points. Intraclass correlations >0.70 were considered acceptable.

Construct validity between SDS scores and reference measures at baseline in the short‐term studies and at open‐label baseline in the maintenance‐of‐efficacy study was assessed using Pearson's *r* (correlation strength: small, 0.10; medium, 0.30; large, 0.50; Cohen, 1988). Known‐groups validity in the two short‐term studies was assessed by stratifying SDS scores at EOS by CGI‐S rating. As previously described for the Y‐BOCS‐BE by Deal et al. (2015), groups were defined as ill (CGI‐S rating ≥4) or not ill (CGI‐S rating 1–3). Analysis of covariance (ANCOVA) models controlling for age and sex assessed between‐group differences in SDS scores, with post hoc comparisons conducted using Scheffe's test.

To assess responsiveness of the SDS to change in the two short‐term studies, SDS total score changes from baseline to EOS were examined in responders versus nonresponders using ANCOVA models that controlled for age, sex, and baseline score. Responders were defined as participants from either treatment group with no binge eating within the last 28 days of the study or with BES scores ≤17 at EOS. Nonresponders were defined as individuals exhibiting binge eating within the last 28 days of the study or with BES scores >17 at EOS.

For SDS responder thresholds in the short‐term studies, score changes indicative of treatment response were identified using triangulation of anchor‐based and distribution‐based methods (Revicki, Hays, Cella, & Sloan, 2008). Criteria for anchor‐based methods included (1) a CGI‐I rating ≤3 at EOS, (2) a ≥2‐point CGI‐S decrease from baseline to EOS, (3) abstaining from binge eating at EOS (defined as 0 binge eating days/week for 4 weeks before EOS), (4) ≤2 binge eating events in any week within the month before EOS, (5) abstaining from binge eating at EOS and a ≥2‐point CGI‐S decrease from baseline to EOS, and (6) having ≤2 binge eating events in any week within the month before EOS and a ≥2‐point CGI‐S decrease from baseline to EOS. Anchor‐based estimates were assessed using Youden's index (sensitivity + specificity − 1).

Criteria for distribution‐based methods included calculating the 0.5 baseline SD value, which is a good approximation of clinically important differences (Norman, Sloan, & Wyrwich, 2003), the 0.5 mean change score SD, and the standard error of measurement.

To assess functional relapse in the maintenance‐of‐efficacy study, the responder threshold was determined by examining SDS scores at week 12/randomized‐withdrawal baseline, week 16, and week 38. To assess functional remission, SDS remission was defined as a total score ≤6 or domain score ≤2 (K. H. Sheehan & Sheehan, 2008; D. V. Sheehan et al., 2011). Remission rates in each group (placebo or LDX) are presented at baseline and week 12 for the short‐term studies and at open‐label baseline, randomized‐withdrawal baseline (week 12), week 16, and week 38 in the maintenance‐of‐efficacy study. For the maintenance‐of‐efficacy study, SDS total score remission rates based on remission status at week 12/randomized‐withdrawal baseline are also reported; data by remission status are not reported for the short‐term studies because these data are published (D. V. Sheehan et al., 2018). For both of these analyses, descriptive data are reported using observed cases.

## 3 RESULTS

### 3.1 Participants

The analyses included 724 participants from the short‐term studies and 267 from the maintenance‐of‐efficacy study. Demographic and clinical characteristics are summarized in Table 1.

**TABLE 1:** Participant demographic and baseline^a^ clinical characteristics

Characteristic	Short‐term studies (*N* = 724)	Maintenance‐of‐efficacy study (*N* = 267)
Mean ± SD age, years	37.8 ± 10.20	38.5 ± 9.93
Female, *n* (%)	627 (86.6)	234 (87.6)
Race, *n* (%)		
White	550 (76.0)	225 (84.3)
Black	129 (17.8)	34 (12.7)
Asian	13 (1.8)	2 (0.7)
Other	31 (4.3)	6 (2.2)
Missing	1 (0.1)	0
Mean ± SD SDS scores		
Total	10.9 ± 7.46^b^	11.1 ± 7.59
Work/school	3.1 ± 2.63^b^	3.2 ± 2.68
Social life/leisure activities	4.2 ± 2.96^c^	4.2 ± 2.87
Family life/home responsibilities	3.7 ± 2.73^c^	3.7 ± 2.80
Mean ± SD binge eating day/week	4.7 ± 1.30	4.8 ± 1.21
Mean ± BES total score	29.0 ± 7.26^d^	N/A
Mean ± SD Y‐BOCS‐BE total score	21.5 ± 4.72^e^	22.4 ± 5.15
Mean ± SD EQ‐5D‐5L scores		
Health status index score	0.9 ± 0.11^c^	0.9 ± 0.13
VAS score	74.1 ± 17.91	173.3 ± 18.57
Mean ± SD CGI‐S rating	4.6 ± 0.68	4.6 ± 0.69

Abbreviations: BES, Binge Eating Scale; CGI‐S, Clinical Global Impressions–Severity; EQ‐5D‐5L, EuroQol Group 5‐Dimension 5‐Level; N/A, not applicable; SDS, Sheehan Disability Scale; VAS, visual analogue scale; Y‐BOCS‐BE=Yale–Brown Obsessive Compulsive Scale modified for binge eating.

^a^ Open‐label baseline in the maintenance‐of‐efficacy study.

^b^ *n* = 720.

^c^ *n* = 722.

^d^ *n* = 723.

^e^ *n* = 721.

### 3.2 SDS psychometric analyses

#### 3.2.1 Item‐level statistics and factor structure

Item‐level descriptive statistics for the SDS and reference measures are summarized in Table 1. Ceiling effects were not observed for SDS total or domain scores at baseline or EOS in the short‐term studies or at open‐label baseline and week 12/randomized‐withdrawal baseline in the maintenance‐of‐efficacy study. In the short‐term studies, floor effects for SDS scores were not observed at baseline but were observed at EOS for SDS total score (48.9% of participants; 338/691) and all SDS domains (work/school: 58.2% of participants [402/691]; social life/leisure activities of participants: 53.3% [369/692]; and family life/home responsibilities: 56.2% [389/692]). Floor effects were not observed at open‐label baseline in the maintenance‐of‐efficacy study, but at week 12/randomized‐withdrawal baseline, floor effects were observed for SDS total score (74.1% of participants; 197/266) and all SDS domains (work/school: 81.6% of participants [217/266]; social life/leisure activities of participants: 80.1% [213/266]; and family life/home responsibilities of participants: 81.2% [216/266]). Confirmatory factor analysis supported a unidimensional SDS total score, with eigenvalues indicating that all SDS domains were good indicators of the construct underlying the SDS (Table 2).

**TABLE 2:** Confirmatory factor loadings for the SDS at baseline^a^

	Constrained family life and social life factor loadings to be equal		Constrained all error variances to be equal	
Eigenvalue (SD)	Short‐term studies (*N* = 722)	Maintenance‐of‐efficacy study (*N* = 267)	Short‐term studies (*N* = 722)	Maintenance‐of‐efficacy study (*N* = 267)
Work/school	0.828 (0.013)	0.829 (0.021)	0.819 (0.012)	0.843 (0.017)
Social life/leisure activities	0.843 (0.014)	0.844 (0.022)	0.867 (0.009)	0.87 (0.014)
Family life/home responsibilities	0.857 (0.015)	0.908 (0.02)	0.84 (0.01)	0.866 (0.014)
	Chi‐square = 42.907 (*p* = 0.0000)	Chi‐square = 5.745 (*p* = 0.0165)	Chi‐square = 15.481 (*p* = 0.0004)	Chi‐square = 7.623 (*p* = 0.0221)
	df = 1	df = 1	df = 2	df = 2
	CFI = 0.964	CFI = 0.99	CFI = 0.988	CFI = 0.988
	RMSEA = 0.241	RMSEA = 0.133	RMSEA = 0.097	RMSEA = 0.103
	90% CI (0.183, 0.305)	90% CI (0.046, 0.247)	90% CI (0.056, 0.144)	90% CI (0.033, 0.184)
	SRMR = 0.081	SRMR = 0.051	SRMR = 0.021	SRMR = 0.022

Abbreviations: CFI, comparative fit index; df, degrees of freedom; RMSEA, root mean square error of approximation; SD, standard deviation; SDS, Sheehan Disability Scale; SRMR, standardized root mean residual.

^a^ Based on open‐label baseline in the maintenance‐of‐efficacy study.

#### 3.2.2 Reliability

Internal consistency (Cronbach's *α*) in the short‐term efficacy studies at baseline was high for SDS total (0.878) and domain (work/school, 0.863; social life/leisure activities, 0.793; and family life/home responsibilities, 0.819) scores. Domain scores were correlated with total score (correlation coefficients: for work/school, 0.723; social life/leisure activities, 0.803; and family life/home responsibilities, 0.773). SDS total and domain scores were significantly different at baseline and week 6 (all *p* < 0.0001), and intraclass correlation coefficients were below the acceptable range (>0.70) for SDS total score (0.529) and all domain scores (work/school, 0.635; social life/leisure activities, 0.467; and family life/home responsibilities, 0.393), indicating that test–retest reliability in the short‐term studies was poor.

#### 3.2.3 Validity

**FIGURE 1:**
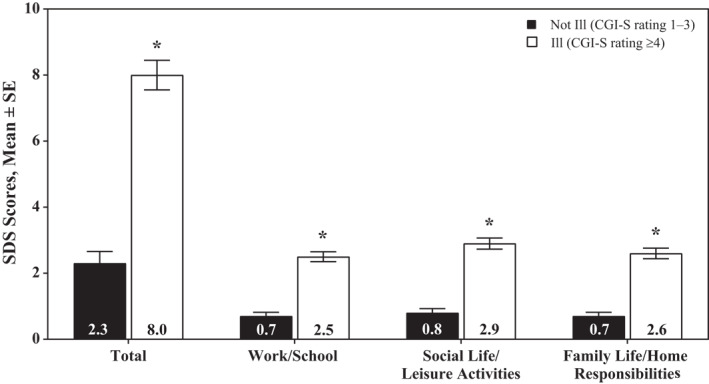
SDS scores at EOS^†^ by CGI‐S group at EOS, short‐term studies. CGI‐S, Clinical Global Impressions–Severity; EOS, end of study; SDS, Sheehan Disability Scale. ^†^Week 12 of treatment. *p < 0.0001

The SDS total scores exhibited good construct validity at baseline in the short‐term studies, with moderate correlations observed for Y‐BOCS‐BE total score, BES score, and EQ‐5D‐5L health status index and VAS scores (Table 3). Similar results were observed in the maintenance‐of‐efficacy study at open‐label baseline. Moderate correlations with SDS total score were observed for Y‐BOCS‐BE total score and EQ‐5D‐5L index scores, and a low correlation was observed for EQ‐5D‐5L VAS score. SDS total score did not correlate with the number of binge eating days/week at baseline in the short‐term studies or maintenance‐of‐efficacy study. Known‐groups validity was demonstrated in the short‐term studies, as measured by significantly lower SDS total (*F*[3657] = 50.21, *p* < 0.0001), work domain (*F*[3657] = 42.13, *p* < 0.0001), social life domain (*F*[3658] = 50.23, *p* < 0.0001), and family life domain (*F*[3658] = 44.61, *p* < 0.0001) scores observed in the not ill (CGS‐S rating, 1–3) versus the ill (CGI‐S rating, ≥4) group at EOS (Figure 1).

**TABLE 3:** Pearson correlations between SDS scores and reference measures at baseline^a^

	Total score		Work/school		Social life/leisure activities		Family life/home responsibilities	
	*r*	*p*‐value	*r*	*p*‐value	*r*	*p*‐value	*r*	*p*‐value
Number of binge eating day/week								
Short‐term studies^b^	0.055	0.1389	0.065	0.0831	0.037	0.3192	0.050	0.1805
Maintenance‐of‐efficacy study^c^	0.072	0.2440	0.103	0.0916	0.059	0.3333	0.034	0.5805
Y‐BOCS‐BE total score								
Short‐term studies^b^	0.350	<0.0001	0.292	<0.0001	0.355	<0.0001	0.290	<0.0001
Maintenance‐of‐efficacy study^c^	0.326	<0.0001	0.289	<0.0001	0.305	<0.0001	0.293	<0.0001
EQ‐5D‐5L health index score								
Short‐term studies^b^	−0.346	<0.0001	−0.291	<0.0001	−0.334	<0.0001	−0.290	<0.0001
Maintenance‐of‐efficacy study^c^	−0.374	<0.0001	−0.286	<0.0001	−0.373	<0.0001	−0.358	<0.0001
EQ‐5D‐5L VAS score								
Short‐term studies^b^	−0.274	<0.0001	−0.222	<0.0001	−0.252	<0.0001	−0.259	<0.0001
Maintenance‐of‐efficacy study^c^	−0.251	<0.0001	−0.174	0.0045	−0.282	<0.0001	−0.225	0.0002
CGI‐S								
Short‐term studies^b^	0.164	<0.0001	0.144	0.0001	0.164	<0.0001	0.124	0.0008
Maintenance‐of‐efficacy study^c^	0.245	<0.0001	0.214	0.0004	0.252	<0.0001	0.201	0.0010
BES								
Short‐term studies^b^	0.355	<0.0001	0.249	<0.0001	0.371	<0.0001	0.342	<0.0001
Maintenance‐of‐efficacy study^c^	N/A		N/A		N/A		N/A	

Abbreviations: BES, Binge Eating Scale; CGI‐S, Clinical Global Impressions–Severity; EQ‐5D‐5L, EuroQol Group 5‐Dimension 5‐Level; N/A, not applicable; SDS, Sheehan Disability Scale; VAS, visual analogue scale; Y‐BOCS‐BE, Yale–Brown Obsessive Compulsive Scale modified for binge eating.

^a^ Based on open‐label baseline in the maintenance‐of‐efficacy study.

^b^ Based on *n* = 724.

^c^ Based on *n* = 267.

#### 3.2.4 Responsiveness to change

**FIGURE 2:**
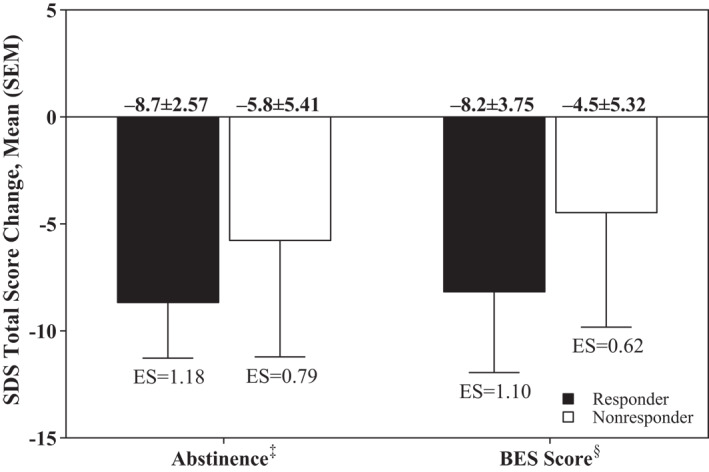
SDS total score change in responders and nonresponders at EOS,^†^ short‐term studies. BES, Binge Eating Scale; EOS, end of study; ES, effect size; SDS, Sheehan Disability Scale. ^†^Week 12 of treatment. ^‡^Responder, no binge eating behavior for the last 28 days of the study; nonresponder, binge eating behavior within the last 28 days of the study. ^§^Responder: BES score ≤17; nonresponder: BES score >17

The magnitude of SDS total score reductions from baseline to EOS in the short‐term studies was significantly greater in responders than nonresponders when response was based on abstinence from binge eating and BES scores (Figure 2).

#### 3.2.5 Responder threshold

Mean ± SD changes in SDS total score from baseline at EOS for anchor‐ and distribution‐based methods in the short‐term studies are summarized in Table 4. Mean ± SD score changes from baseline to EOS for domain scores ranged from −2.0 ± 2.56 to −2.4 ± 2.63 for work/school, −2.8 ± 2.88 to −3.3 ± 2.92 for social life/leisure activities, and −2.5 ± 2.76 to 2.9 ± 2.71 for family life/home responsibilities. For anchor‐based cutoffs, Youden's index suggested optimal score reduction cutoffs of 6–7 points for SDS total score, 1.5–2 points for work/school and family life/home responsibilities scores, and 2–3 points for social life/leisure activities scores. Distribution‐based estimates were lower, with the 0.5 mean change score SD estimate being 3.76 (0.5 baseline SD = 3.72) for SDS total score (Table 4). The 0.5 mean change score SD estimates were 1.33 (0.5 baseline SD = 1.32) for work/school, 1.47 (0.5 baseline SD = 1.48) for social life/leisure activities, and 1.43 (0.5 baseline SD = 1.36) for family life/home responsibilities. Based on the findings of the anchor‐based and distribution‐based methods, the threshold for change to responder status was estimated to be ≥4 points for SDS total score and ≥2 points for domain scores for BED.

**TABLE 4:** Responder thresholds for SDS total score, short‐term studies

Responder threshold	*n*	Mean ± SD	ROC analysis	YI
Anchor‐based cutoffs				
CGI‐I ≤3 at EOS	545	−7.5 ± 7.31	≤−5	0.29
≥2‐point CGI‐S decrease from baseline to EOS	414	−8.2 ± 7.48	≤−6 and ≤−9	0.22
Abstinence from binge eating at EOS	185	−8.4 ± 7.42	≤−6	0.15
Abstinence from binge eating at EOS *and* ≥2‐point CGI‐S decrease from baseline to EOS	177	−8.7 ± 7.35	≤−6	0.17
≤2 binge eating events in any week within a month before EOS	553	−7.4 ± 7.18	≤−4	0.26
≤2 binge eating events in any week within a month before EOS *and* ≥2‐point CGI‐S decrease from baseline to EOS	399	−8.3 ± 7.31	≤−6	0.17
Distribution‐based cutoffs				
0.5 baseline SD	724	3.72	–	–
0.5 mean change score SD	724	3.76	–	–
Standard error of measurement	724	5.39	–	–

Abbreviations: CGI‐I, Clinical Global Impressions–Improvement; CGI‐S, Clinical Global Impressions–Severity; EOS, end of study (defined as week 12 of treatment); ROC, receiver operating characteristic; SD, standard deviation; SDS, Sheehan Disability Scale; YI, Youden's index.

#### 3.2.6 Functional relapse and functional remission

**FIGURE 3:**
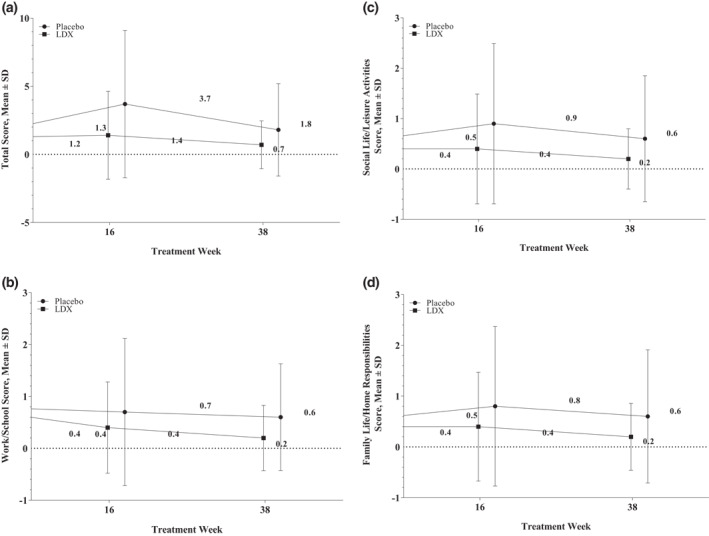
Scores for SDS (a) total, (b) work/school, (c) social life/leisure activities, and (d) family life/home responsibilities, maintenance‐of‐efficacy study.^†^ LDX, lisdexamfetamine dimesylate; RWB, randomized‐withdrawal baseline; SDS, Sheehan Disability Scale. ^†^Sample size: total score (baseline: placebo [*n* = 131 week 12/RWB, *n* = 124 at week 16, *n* = 54 at week 38], LDX [*n* = 136 week 12/RWB, *n* = 134 at week 16, *n* = 108 at week 38]); domain scores (baseline: placebo [*n* = 131 week 12/RWB, *n* = 93 at week 16, *n* = 50 at week 38], LDX [*n* = 136 week 12/RWB, *n* = 126 at week 16, *n* = 102 at week 38])

At week 12/randomized‐withdrawal baseline of the maintenance‐of‐efficacy study, mean SDS scores were comparable between participants randomized to placebo or LDX (Figure 3). In participants randomized to placebo, mean ± SD total and domain scores increased relative to week 12/randomized‐withdrawal baseline at weeks 16 and 38. In contrast, mean ± SD total and domain scores were unchanged at week 16 and decreased at week 38 relative to week 12/randomized‐withdrawal baseline in participants randomized to LDX.

**FIGURE 4:**
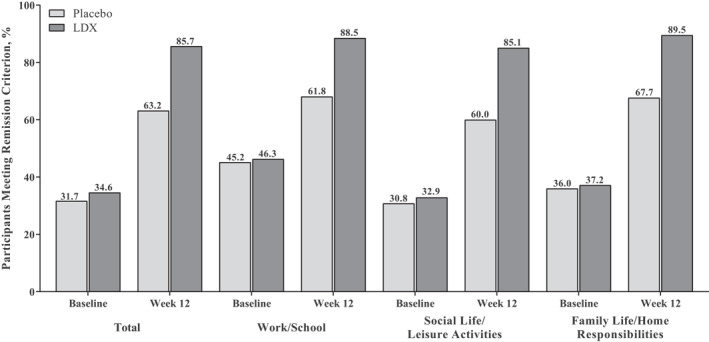
Percentage of participants meeting remission criterion, short‐term studies.^†^ LDX, lisdexamfetamine dimesylate. ^†^Missing values: baseline (placebo: total and work/school [*n* = 2 each], social life/leisure activities and family life/home responsibilities [*n* = 1 each]; LDX: total and work/school [*n* = 2 each], social life/leisure activities and family life/home responsibilities [*n* = 1 each]); week 12: (placebo: total and all subscales [*n* = 55 each]; LDX: total and work/school [*n* = 47 each], social life/leisure activities and family life/home responsibilities [*n* = 46 each])

**FIGURE 5:**
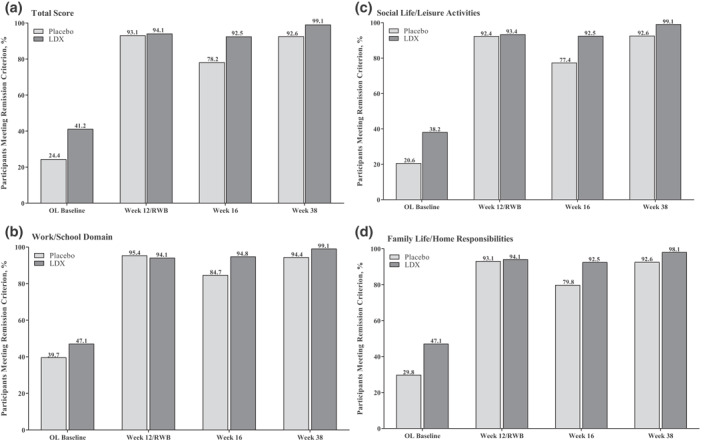
Percentage of participants meeting remission criterion, maintenance‐of‐efficacy study.^†‡^ LDX, lisdexamfetamine dimesylate; OL, open‐label; RWB, randomized‐withdrawal baseline. ^†^Missing values: open‐label baseline (placebo [*n* = 0], LDX [*n* = 0]); RWB (placebo [*n* = 0], LDX [*n* = 0]); week 16 (placebo [*n* = 7]; LDX [*n* = 2]; week 38 [placebo [*n* = 77], LDX [*n* = 28]). ^‡^Dichotomization of placebo and LDX at open‐label baseline is based on randomization group at the RWB

In the short‐term efficacy studies, <50% of participants met SDS remission criteria at baseline (Figure 4). Remissions rates were roughly comparable with LDX and placebo at baseline, but the percentages of participants meeting remission criteria were greater with LDX than placebo at week 12 (Figure 4). Similarly, <50% of participants met SDS remission criteria at open‐label baseline in the maintenance‐of‐efficacy study (Figure 5). However, >90% of participants in both treatment groups met SDS remission criteria at week 12/randomized‐withdrawal baseline (Figure 5a‐5d). Following randomization, the percentage of participants meeting SDS remission criteria decreased from week 12/randomized‐withdrawal baseline to week 16 and then increased from weeks 16 to 38 with placebo (Figure 5a–5d). In participants randomized to LDX, remission rates remained relatively stable from week 12/randomized‐withdrawal baseline to week 38 (Figure 5a–5d).

The SDS total score remission rates as a function of week 12/randomized‐withdrawal baseline remission status are summarized in Table 5. Most participants who met remission criteria at week 12/randomized‐withdrawal baseline did not meet remission criteria at open‐label baseline but did meet remission criteria at weeks 16 and 38. None of the participants who did not meet remission criteria at week 12/randomized‐withdrawal baseline met remission criteria at open‐label baseline, and most did not meet remission criteria at week 16.

**TABLE 5:** Percentage of participants meeting SDS total score remission criterion by remission status,^a^ maintenance‐of‐efficacy study

	Remission, *n*/*N* (%)		Nonremission, *n*/*N* (%)		Missing, *n* ^b^	
	Placebo	LDX	Placebo	LDX	Placebo	LDX
Remission at week 12/RWB						
OL baseline	32/122 (26.2)	56/128 (43.8)	90/122 (73.8)	72/128 (56.3)	0	0
Week 12/RWB	122/122 (100)	127/127 (100)	0	0	0	1
Week 16	96/115 (83.5)	121/126 (96.0)	19/115 (16.5)	5/126 (4.0)	7	2
Week 38	48/52 (92.3)	102/102 (100)	4/52 (7.7)	0	70	26
Nonremission at week 12/RWB						
OL baseline	0	0	9/9 (100)	8/8 (100)	0	0
Week 12/RWB	0	0	9/9 (100)	8/8 (100)	0	0
Week 16	1/9 (11.1)	3/8 (37.5)	8/9 (88.9)	5/8 (62.5)	0	0
Week 38	2/2 (100)	5/6 (83.3)	0	1/6 (16.7)	7	2

Abbreviations: LDX, lisdexamfetamine dimesylate; OL, open‐label; RWB, randomized‐withdrawal baseline; SDS, Sheehan Disability Scale.

^a^ Remission is defined by SDS total score at week 12/randomized‐withdrawal baseline (remission, ≤6 and nonremission, >6).

^b^ Number of participants with missing scores.

## 4 DISCUSSION

The key findings of these analyses are that the SDS demonstrated good internal consistency (Cronbach's *α* > 0.70) and validity, was responsive to change, and exhibited stability with continued treatment in adults with BED. Based on anchor‐based and distribution‐based estimation methods for meaningful change, reductions of ≥4 points for SDS total score and ≥2 points for SDS domain scores were found to represent improvement to “response” status in these LDX clinical trials.

The overall findings of the psychometric analyses were comparable with previous reports in other populations (Arbuckle et al., 2009; Coles et al., 2014; Leon et al., 1992, 1997). The unidimensional factor structure observed in individuals with BED is consistent with previous observations in individuals from a primary care setting (Leon et al., 1997), individuals diagnosed with bipolar disorder (Arbuckle et al., 2009), and individuals diagnosed with panic disorder (Leon et al., 1992). The levels of internal consistency, as measured by Cronbach's *α*, and of inter‐item correlations were also within ranges observed in other published reports (Arbuckle et al., 2009; Coles et al., 2014; Leon et al., 1992, 1997).

In the current study, poor test–retest reliability was observed at baseline and week 6 in the short‐term studies when assessing a subset of participants receiving placebo considered clinically stable based on unchanged CGI‐S ratings. These findings may be related to using a long test–retest period and defining clinical stability based on the CGI‐S. In a study of adults diagnosed with ADHD, the threshold for good test–retest reliability was met for SDS total score (intraclass correlation coefficient = 0.72) when assessed over a 9‐week period; however, intraclass correlation coefficients for the SDS domains were lower (range: 0.59–0.69; Coles et al., 2014). In individuals diagnosed with bipolar disorder, good test–retest reliability was reported over a 5‐ to 10‐day period for SDS total scores and social life/leisure activities domain scores (intraclass correlation coefficients ≥0.70), with lower intraclass coefficients observed for the family life/home responsibilities and work/school domains (0.61 and 0.63, respectively; Arbuckle et al., 2009).

In this population of individuals with BED, SDS total and domain scores demonstrated construct validity in reference to binge eating (Y‐BOCS‐BE and BES scores), quality of life (EQ‐5D‐5L index and VAS scores), and overall disease severity (CGI‐S) at baseline. However, SDS scores did not correlate with binge eating frequency. The reason for this is unclear. It could be related to variability in the level of binge eating perceived to be impairing. Some individuals may have frequent mild binge eating episodes that are not very disabling, whereas others may exhibit a low frequency of extended binge eating episodes that are highly disabling. Therefore, there is a loss of sensitivity for frequency‐related measures. Known‐groups validity was demonstrated, as measured by significant differences in SDS scores in individuals categorized as ill versus not ill at EOS. Taken together, these findings support the reliability and validity of the SDS in individuals with BED.

The baseline levels of functional disability as measured by SDS scores in this population were roughly comparable to those observed in the primary care setting and in individuals diagnosed with panic disorder (Leon et al., 1992, 1997), but were lower compared with individuals diagnosed with ADHD or bipolar disorder (Arbuckle et al., 2009; Coles et al., 2014; Weiss et al., 2012). Based on the mean SDS total score at baseline, the level of functional impairment in this population of individuals with BED was mild to moderate. However, moderate to large effect sizes for the change from baseline to EOS in SDS total score were observed in responders and nonresponders, with the reported effect sizes observed in treatment responders being comparable with those observed in individuals with panic disorder treated with alprazolam (Leon et al., 1992).

Anchor‐ and distribution‐based methods estimated that reductions of ≥4 points for SDS total score and ≥2 points for domain scores represented response in this BED population. These thresholds, which are consistent with the distribution‐based values and lower than the anchor‐based Youden's index, are similar to previously reported responder definitions (Arbuckle et al., 2009; Coles et al., 2014; K. H. Sheehan & Sheehan, 2008). In individuals diagnosed with bipolar disorder, mean changes considered to be “minimally improved” were estimated to be 6.0 points for total score (domain scores = 1.38–2.34 points), and 0.5 SD was estimated to be 4.05 points for total score (domain scores = 1.41–1.59 points; Arbuckle et al., 2009). In individuals diagnosed with ADHD, the responder thresholds for SDS total score were slightly lower, with anchor‐based methods estimating a mean change of 2.53 points and distribution‐based methods estimating the 0.5 SD to be 2.75 points (Coles et al., 2014). In the current analyses, large differences in were observed between the anchor‐ and distribution‐based methods. Because a majority of participants achieved treatment response based on the anchor‐based analyses, the decision was made to focus on the distribution‐based values to define response. This helped differentiate response from the well‐established remission threshold (K. H. Sheehan & Sheehan, 2008; D. V. Sheehan et al., 2011). Additionally, the lower Youden's index values indicate that estimates from the anchor‐based methods may not be the best values to establish the minimal important difference or treatment response.

When assessing stability of SDS scores in the randomized‐withdrawal study, it was observed that SDS scores increased compared with baseline at week 16 and EOS to a greater extent in participants randomized to placebo than to LDX. In participants randomized to placebo, SDS total score increased by 2.51 points and by <1 point for the SDS domain scores (work/school: 0.88; social life/leisure activities, 0.80; and family life/home responsibilities, 0.83). There are no guidelines for BED relapse. However, compared with the estimated responder thresholds established based on anchor‐based and distribution‐based methods in the short‐term studies (total score change ≥4 points and domain score changes ≥2 points), these findings suggest that SDS scores did not change by a meaningful amount during the randomized‐withdrawal phase of this study.

Assessment of functional remission based on SDS scores indicated that a substantial percentage of participants in the short‐term efficacy studies (approximately 65%–70%) and the maintenance‐of‐efficacy study (approximately 60%–80%) exhibited functional disability at baseline. Furthermore, LDX treatment was associated with numerically greater SDS remission rates than placebo in the short‐term efficacy studies at week 12 and with sustained remission rates (>90%) during the randomized‐withdrawal phase of the maintenance‐of‐efficacy study. However, comparisons between LDX and placebo at weeks 16 and 38 of the randomized‐withdrawal phase of the maintenance‐of‐efficacy study should be interpreted cautiously because of sample size differences resulting from discontinuation due to relapse.

These data should be considered in light of certain limitations. First, study participants did not have comorbid illnesses or psychiatric conditions. As individuals with BED are at increased risk of having medical and psychiatric comorbidities that can affect quality of life and functioning (D. V. Sheehan & Herman, 2015), it is not known how these findings would translate to a more heterogeneous population of individuals with BED. Second, substantive floor effects were observed at EOS for SDS total and domain scores. As LDX demonstrated strong treatment effects on multiple study endpoints, the observed floor effects may be partially explained by LDX treatment effects. Third, stability and remission rate findings reported during the randomized‐withdrawal phase of the maintenance‐of‐efficacy study should be interpreted cautiously because the differential relapse rates between treatment groups—32.1% with placebo versus 3.7% with LDX (Hudson et al., 2017)—may have biased the results. Finally, as noted previously, the poor test–retest reliability observed in these analyses is likely attributable to factors related to the use of a longer test–retest period than is typical used for test–retest assessments and to the use of the CGI‐S to define clinical stability.

In conclusion, in adults with moderate to severe BED who participated in LDX clinical trials, the SDS demonstrated good internal consistency and validity, was responsive to change, and exhibited stability with continued LDX treatment. Anchor‐based and distribution‐based methods estimated that improvement in functional disability to responder status in adults with BED is reflected by a change of ≥4 points on the SDS total score and ≥2 points on the individual domain scores.

## CONFLICTS OF INTEREST

Karen S. Yee is an employee of Shire, a member of the Takeda Group of Companies, Cambridge, MA and holds Takeda stock and/or stock options. Robin Pokrzywinski, Asha Hareendran, and Shannon Shaffer are employees of Evidera, which was funded by Shire to conduct these analyses. David V. Sheehan is a consultant to Shire and the author of the Sheehan Disability Scale, which measures functional impairment and is the focus of this manuscript.
